# Vaccine Efficacy on the Novel Reassortant H9N2 Virus in Indonesia

**DOI:** 10.3390/vaccines8030449

**Published:** 2020-08-10

**Authors:** Ni Luh Putu Indi Dharmayanti, Risa Indriani, Diana Nurjanah

**Affiliations:** Indonesian Research Center for Veterinary Science, Bogor 16114, Indonesia; risain52@yahoo.com (R.I.); diananurjanah@gmail.com (D.N.)

**Keywords:** avian influenza, reassortant, inactivated, vaccine, pathogenicity, efficacy

## Abstract

Vaccination is one of the leading methods of controlling the spread of the Avian Influenza (AI) viruses in Indonesia. The variety of circulating viruses and their ability to mutate must be followed by updating the vaccine master seed used in the field. In this study, we identified the reassortant H9N2 viruses in chicken farms that showed significant problems in decreased egg production with high mortality. The reassortant H9N2 viruses derived the PB2 gene from the H5N1 virus. The pathogenicity test results of the reassortant virus showed various clinical signs of illness, a high mortality rate (10%), and decreased egg production down to 63.12% at two weeks post-infection. In a vaccine efficacy test, the vaccinated groups showed minimally decreased egg production that started to increase to more than 80% at 4–7 weeks post-challenge. Our study showed that inactivated bivalent and monovalent reassortant H9N2 vaccines can induce antibody response, reducing the mortality and virus shedding caused by reassortant H9N2 virus infection. The reassortant H9N2 virus is a threat that requires vigilance in poultry farms and the industry. The vaccines used in this study can be one of the options for control or prevention measures on farms infected with the reassortant H9N2 viruses.

## 1. Introduction

Avian Influenza (AI) is a type A influenza virus that belongs to the *Orthomyxoviridae* family. The AI virus genome is a negative-sense single-stranded RNA with eight segments that encode 10 proteins including Polymerase Acidic (PA), Polymerase Base (PB1 and PB2), Nucleoprotein (NP), Hemagglutinin (HA), Neuraminidase (NA), Matrix (M1 and M2) and Non-Structural (NS1 and NS2). Influenza A subtypes can be categorized based on these HA and NA genes. Meanwhile, according to molecular characteristics and pathogenicity in causing disease and death in chickens, AI viruses can be divided into Highly Pathogenic Avian Influenza (HPAI) and Low Pathogenic Avian Influenza (LPAI) [1].

Reassortment is a mechanism for genetic changes in influenza viruses that can occur through the exchange of gene segments by two or more different types of influenza viruses. Genetic reassortment can produce new variants/strains or subtypes that may have different biological characteristics from virus ancestral [2,3]. The reassortment process can occur if two different virus strains infect the same cell simultaneously (co-infection) [4]. The segmented genome structure of influenza A viruses allows the exchange of gene segments when viral co-infection occurs in a cell that can create stable reassortant viruses [3,5].

The reassortant H5N1 virus (Pessel/BPPVRII/07) that has the NS gene from the H3N2 subtype virus was first reported in Indonesia in 2011 [6]. Furthermore, in 2018, a reassortant event was reported between the HPAI H5N1 and LPAI virus, which has a constellation of PB1, PB2, and PA genes derived from the LPAI virus [7]. In 2020, the reassortment between the H5N1 virus clade 2.3.2 and clade 2.1.3 has been found circulating in the live bird markets in Indonesia [8].

The H9N2 virus is an LPAI virus that is endemic in Europe, Asia, and Africa and has been found to be capable of infecting humans and other mammals such as pigs [9]. This virus was first isolated in 1966 from turkeys in the USA and was first discovered infecting humans in 1998 in China [10,11,12]. In Indonesia, the LPAI H9N2 virus has been circulating since 2016 and has caused reduction in the quality and quantity of egg production as well as increased mortality [13,14]. Chickens with a single H9N2 infection show clinical signs of weight loss, irregular egg shape with thin shells, and massive bleeding in the ovaries, oviduct, and other vital organs. Decreased egg production might be caused by the process of replication of this virus in the reproductive tract (infundibulum), causing lesions from acute to chronic [15,16].

In addition, H9N2 virus was found to be an internal gene donor for several AI virus subtypes, including the HPAI H5N1 virus that has been dominant in Indonesia since 2003 [17,18]. Co-circulation of H9N2 and H5N1 viruses in the field will increase the chance of reassortment of genes between the viruses. The reassortant viruses between H9N2 and H5N1 were found to have higher pathogenicity and produce more efficient transmission among mammals [19].

Circulation of the H9N2 and H5N1 viruses as well as the possibility of reassortment between the two viruses in Indonesia has led to different situations in virus control and vaccine application in the field. Vaccination is a method that has so far been effective in controlling the spread of AI viruses in Indonesia. Outbreaks of diseases that arise often cause severity due to co-infection between AI viruses or other viruses such as Newcastle Disease Virus (NDV), Infectious Bronchitis Virus (IBV), bacterial infections, and the possibility of reassortant viruses between the H5N1 and H9N2 viruses.

In this study, we identified a reassortant virus between the H9N2 and H5N1 viruses, which cause a high mortality on the chicken farm. We also tested the pathogenicity of the reassortant viruses and the efficacy of the vaccines against the reassortant H9N2 viruses.

## 2. Materials and Methods

### 2.1. Virus Isolation and Propagation

In this study, two AI viruses, namely the H9N2 subtype (A/chicken/East Java/BLi25Ut/2018) was isolated in 2018 from the farm that has experienced respiratory diseases, decreased egg production, and high mortality rate (>10%) in Blitar district, East Java, Indonesia and also A/chicken/Central Java/SLO.105/2018 isolates obtained from surveillance at the live birds market in Central Java, Indonesia. Based on our genetic analysis, both viruses are reassortant H9N2 viruses. The isolates were propagated in specific pathogen-free (SPF) embryonated chicken eggs 9–11 days old [20]. Furthermore, the pathogenicity and vaccine efficacy tests were carried out using BLi25Ut/18 isolate.

### 2.2. Extraction of Viral RNA, RT-PCR

Viral RNA from infected allantoic fluid was extracted using QIAmp RNA Mini kits (Qiagen, Hilden, Germany) according to the manufacturer’s instructions. RT-PCR reaction was performed with a 9800 Fast Thermal Cycler Applied Biosystems machine (Qiagen, Hilden, Germany) using Superscript III One-Step RT-PCR system by Life Technologies (Waltham, MA, USA) with reaction mixture 10 µL RNA as the template, 2 µL for each primer, 1 µL Taq Polymerase enzyme, 25 µL PCR Master Mix (2X), and NFW (nuclease-free water) up to 50 µL. RT-PCR was performed using the thermal cycle conditions as follows: initial denaturation at 42 °C for 45 min; 95 °C for 3 min; denaturation at 95 °C for 30 s; annealing at 50 °C for 40 s; extension at 72 °C for 40 s (35 cycles); and final extension at 72 °C for 10 min. RT-PCR amplification in the DNA sequencing process was carried out in all viral genomes, namely the HA, NA, NS, M, PB1, PB2, NP, and PA genes.

The amplicon was separated with 1.0% agarose gel electrophoresis and visualized with a transilluminator. H9N2 primers used in this study to amplify HA, NA, M, NS, PB1, PA and NP gene were designed by the author, while the PB2 gene primer Supplementary Materials was designed according to Dharmayanti et al. (2018;2014) [7,21]. Supplementary Materials DNA primers are available as supplemental data.

### 2.3. DNA Sequencing, Virus Characterization, and Phylogenetic Tree

The specific band was purified with the QIAquick Gel Purification System (Qiagen, Hilden, Germany). The results of DNA purification were continued in two-way Direct Sequencing with an ABI PRISM BigDye Terminator Cycle Sequencing Ready Reaction Kit 2.0 (Applied Biosystem, Foster City, CA, USA) in the 3130 Genetic Analyzer (Applied Biosystems, Foster City, CA, USA) machine. The sequencing results were verified and edited using BioEdit Version 7.1.3.0. The Supplementary Materials data of the viruses were identified by Basic Local Alignment Search Tool (BLAST) analysis (http://www.ncbi.nlm.nih.gov). The phylogenetic tree was generated using the MEGA 5.2. Software package (www.megasoftware.net). Each phylogenetic analysis was tested using a bootstrap analysis with 1000 bootstrap replicates. The evolutionary histories of the viruses were inferred using a maximum likelihood calculation based on the Tamura–Nei model. The genome Supplementary Materials of BLi25Ut/18 and SLO.105/18 viruses have been deposited in the Global Initiative on Sharing All Influenza Data (GISAID) EpiFlu database under the isolate ID EPI_ISL_464091 and EPI_ISL_464095, respectively.

### 2.4. Pathogenicity Study of AI H9N2 Virus (BLi25Ut/18)

A Hemagglutination Inhibition (HI) test using specific sera was conducted to confirm that the BLi25Ut/18 isolate has no contamination with other viruses. Specific serum was obtained from the SPF chicken’s serum in the first vaccination with H9N2/2017, H5N1 Clade 2.1.3, H5N1 Clade 2.3.2, ND (Newcastle Disease) genotype VII, ND genotype VI, and ND RIVs vaccines and collected 2 weeks post-vaccination. The animal experiment was approved by the Indonesian Agency for Agricultural Research and Development (IAARD), Institutional Animal Care and Use Committee (IACUC) with registration number Balitbangtan/BBLitvet/A/04/2019. The pathogenicity test of the BLi25Ut/18 virus was conducted in BSL-3 Laboratory and measured by infecting 23-week-old SPF chickens. Ten chickens were infected with 10^6^ EID_50_ BLi25Ut/18 virus per 100 µL individually by intranasal, and the other 10 chickens were infected with 100 µL PBS as control. Each group was kept in an isolator cage and observed every day for 14 days to monitor the clinical signs and egg production. At the end of the experiment, internal organs (lung, heart, spleen, liver, and ovaries) from all chicken groups were collected for gross anatomy and histopathological examination to determine the severity of the lesions. The collected organs were fixed in 10% buffered formalin for at least 24 h, processed routinely, and embedded in paraffin. The paraffin-embedded tissues were sectioned into 2.5-µm-thick sections and stained with hematoxylin and eosin (H&E).

### 2.5. Vaccine Efficacy Test

This study was conducted using 40 SPF chickens that kept until the egg production reached its peak (age 23 weeks) in laboratory BSL-3 cages. The chickens were fed and drank with the ad libitum method. The 23-week-old chickens were randomly selected and divided into four groups; each group consisted of 10 chickens. Group 1 was vaccinated with inactivated bivalent HPAI H5N1/2013 (A/muscovy duck/Banten/BR7/2013) and LPAI H9N2/2017 (A/chicken/West Java/BBLitvet-RI/2017) vaccine (Patent IDP000056903) [22] and challenged with BLi25Ut/18 virus. Group 2 received inactivated monovalent H9N2 vaccine using BLi25Ut/18 virus as master seed and challenged with BLi25Ut/18 virus. Group 3 was unvaccinated and challenged with BLi25Ut/18 virus. Group 4 was unvaccinated and non-challenge (Control Group). For vaccines preparation, antigens were inoculated in 10-day-old SPF embryonated chicken eggs for further propagation of the virus. The 50% embryo infectious dose (EID_50_) was determined by the Reed–Muench method [23]. The antigens were inactivated by β-propiolacton (1:3000) and emulsified in ISA 70VG Montanide™ (at a 30:70 ratio). The doses given in every chicken are 0.5 mL of bivalent and monovalent vaccines intramuscularly. The antigen mass in every single dose in each vaccine is 1280 HAU (Hemagglutination Unit). HI assay was conducted to measure the response of vaccination and antibody titer following the Office International des Epizooties (OIE) procedure [20]. The titer of challenge virus, BLi25Ut/18 virus, is 10^6^ EID_50_ dose/chicken by intranasal/ocular. The challenge chickens were observed in the morning and evening to monitor the clinical signs. Shedding virus was measured by inoculation of the cloacal swab of the chickens group infected with BLi25Ut/18 virus on 4, 8, and 14 days post-infection in 10-day-old embryonated SPF chicken eggs. The inoculated eggs were incubated at 37 °C and observed daily. After 72–96 h, the allantoic fluid was harvested aseptically. The allantoic fluid was tested using 10% normal chicken red blood cells (RBC) to find out the hemagglutinin agent. Observation of chicken egg production post-challenge was measured by the average number of eggs laid per week, the number of egg production per week/number of chicken population x 100%. Serological data analysis was presented with a geometric mean titer (GMT).

### 2.6. Statistical Analysis

IBM SPSS Statistics 25 software (IBM, Armonk, NY, USA) was used for statistical analysis. Egg production in 2 weeks p.i (post-infection) between the infected and control group was analyzed using a one-way ANOVA test followed by Duncan’s test. *p* Values of <0.05 were considered statistically significant.

## 3. Results

### 3.1. Reassortant Virus Analysis

**Figure 1:**
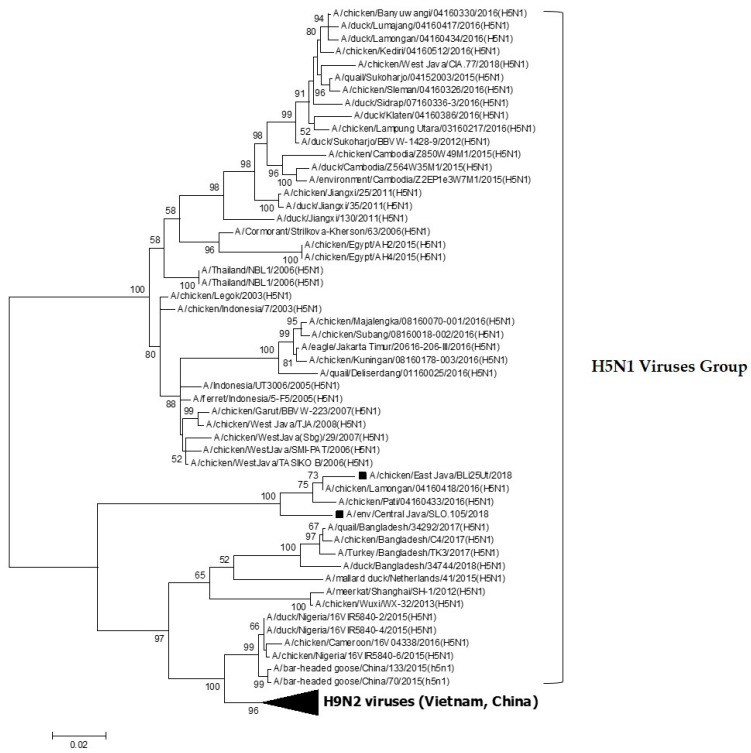
Phylogenetic tree of the PB2 gene of A/chicken/East Java/BLi25Ut/2018 and A/chicken/Central Java/SLO.105/2018 viruses are shown with black box marks. Both isolates are reassortant H9N2 influenza viruses containing PB2 genes from H5N1.

**Figure 2:**
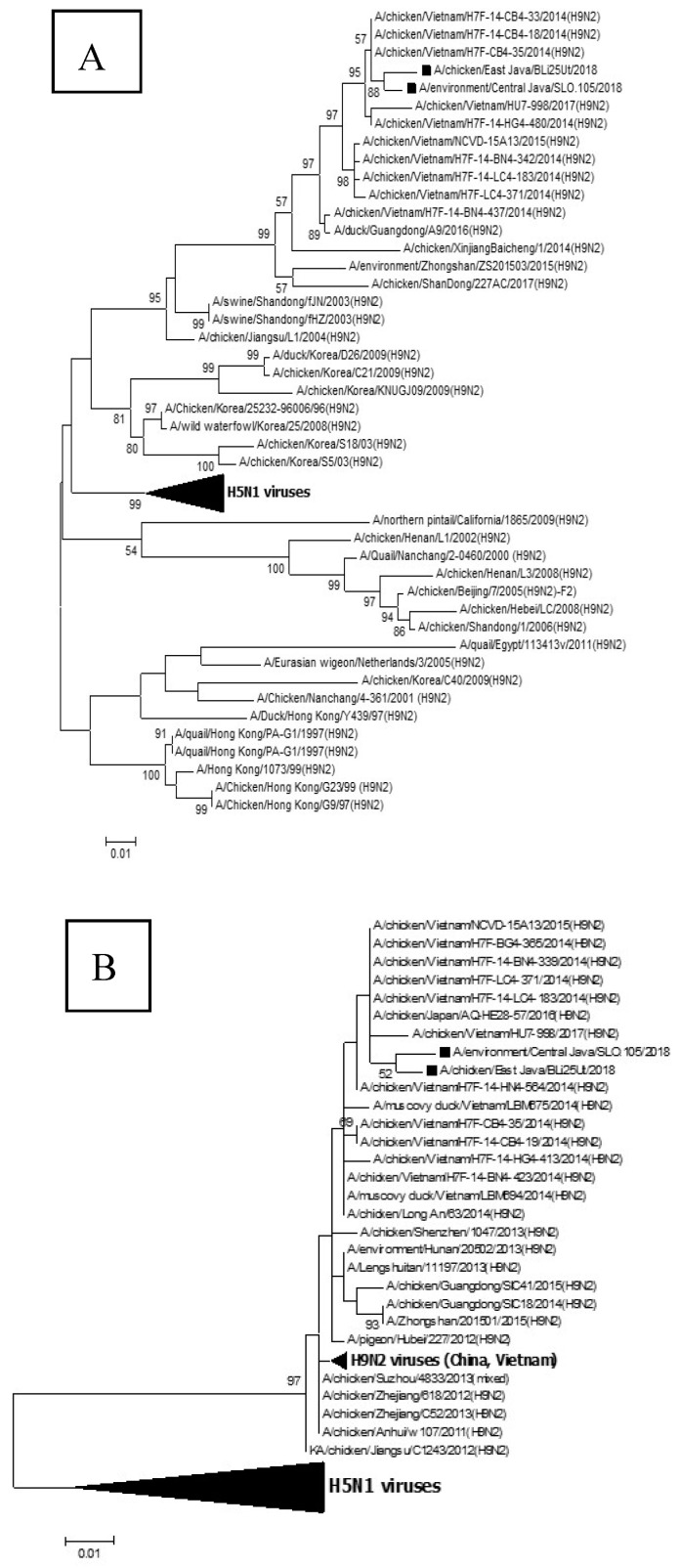

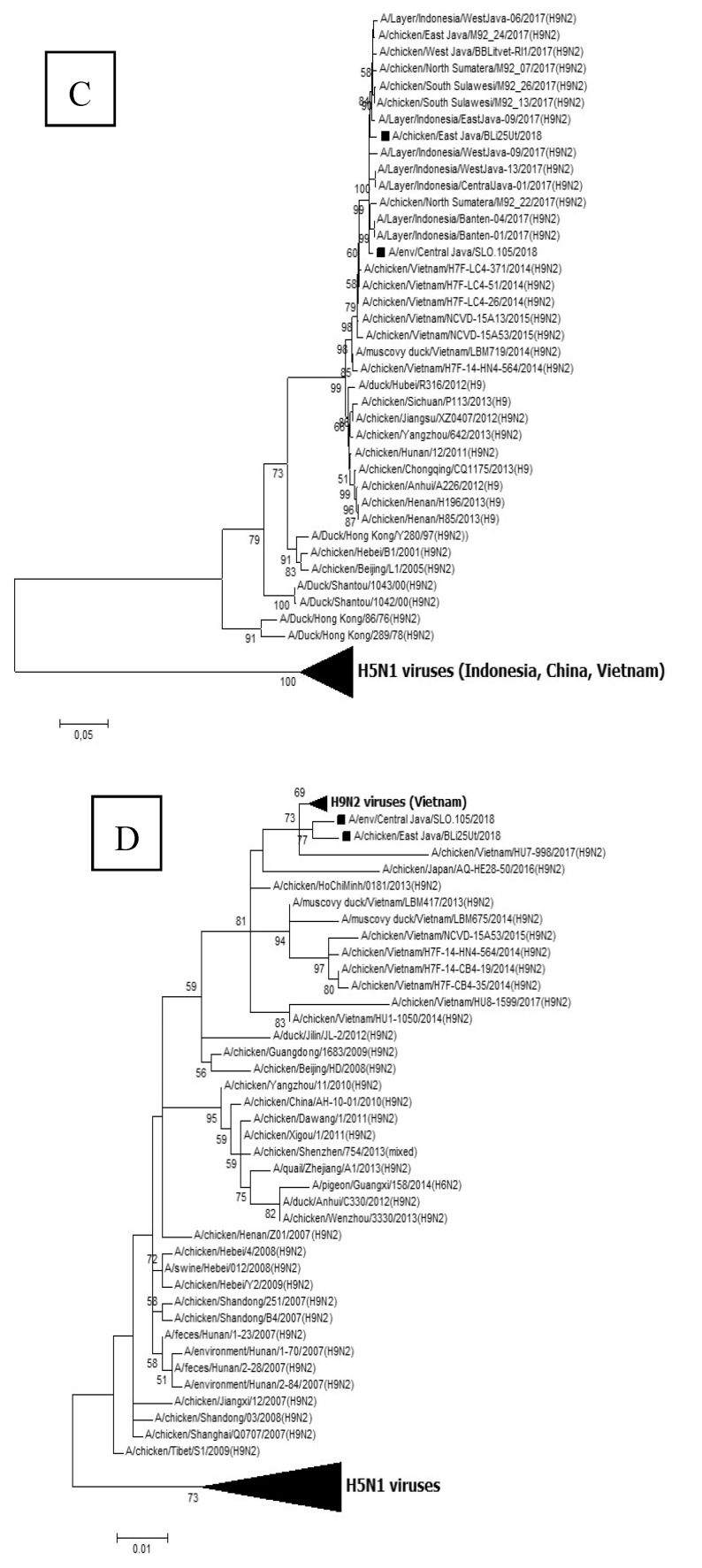

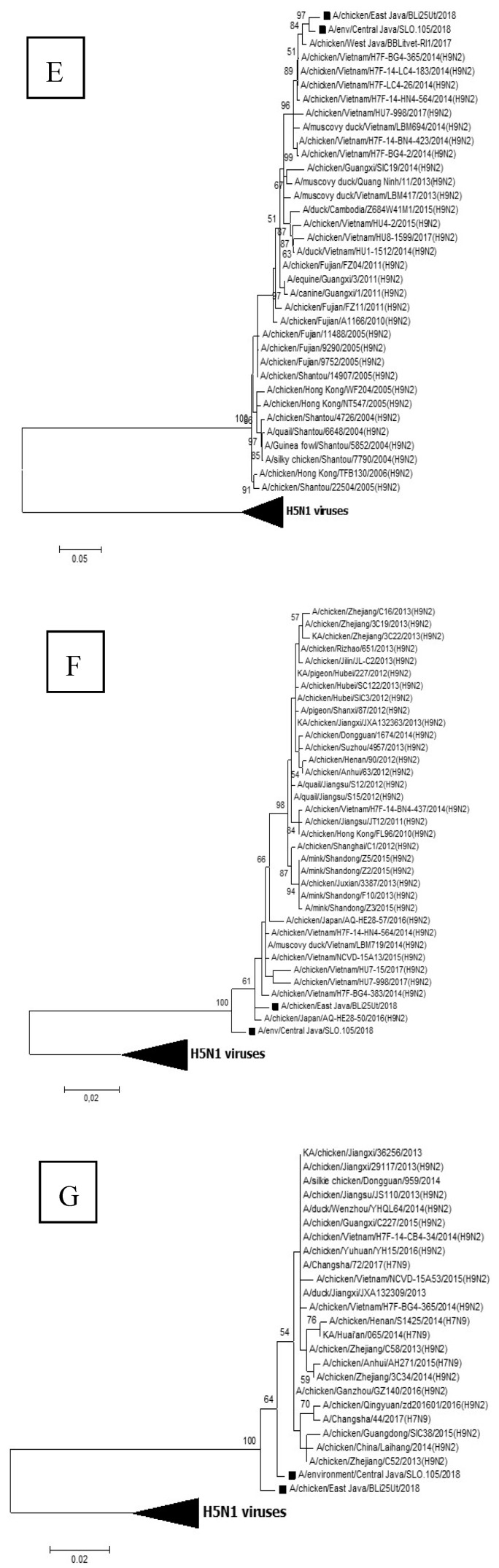
The phylogenetic trees of PB1 (**A**), PA (**B**), HA (**C**), NP (**D**), NA (**E**), M (**F**), and NS (**G**) genes from the H9N2 viruses. The viruses used in this study are shown with black box marks. PA: Polymerase Acidic, HA: Hemagglutinin, NP: Nucleoprotein, NA: Neuraminidase M: Matrix, NS: Non-Structural.

The results of the phylogenetic analysis showed that BLi25Ut/18 and SLO.105/18 isolates, which are the viruses, are suspected of having reassortment with the HPAI H5N1 virus in the PB2 gene (Figure 1) and one group with other Indonesian H5N1 viruses. The PB2 gene derived from the HPAI H5N1 virus will certainly cause changes in the biological character of the LPAI reassortant H9N2 virus. The other genes, i.e., PB1, PA, HA, NP, NA, M and NS, belong to the H9N2 viruses (Figure 2A–G). The viruses characterized in this study have close genetic relationships with the Vietnamese H9N2 viruses and most of the Indonesian H9N2 viruses.

### 3.2. Pathogenicity Study of BLi25Ut/18 Virus

The SPF embryonated chicken eggs that were infected with the BLi25Ut/2018 virus showed several lesions while embryonic death occurred after ± 96 h post-infection. The results of the HI assay using specific sera to ensure that BLi25Ut/18 virus has no contamination with other viruses are presented in Table 1.

**Figure 3:**
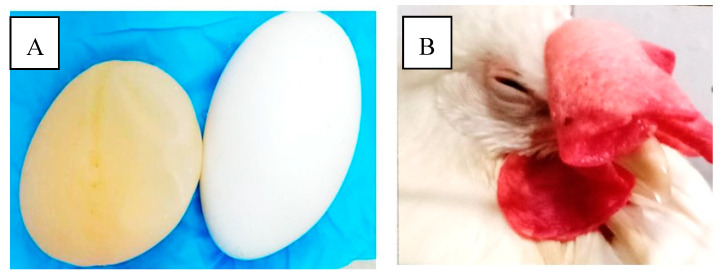
Clinical signs appeared at 4 and 5 days post-infection (p.i.). (**A**) Infected chickens were characterized by laying deformed or soft-shelled eggs (**left**) compared with that of the control group (**right**). (**B**) Clinical signs following infection of chickens with BLi25Ut/18 virus include lethargy and decreased appetite.

The results of the pathogenicity test (Table 2) showed the clinical changes as soft eggshells occurred at 5 days post-infection (Figure 3) when compared with the control group. The pathogenicity has been observed for 14 days post-infection. Clinical signs started to occur in all chickens in infected group at 4 days post-infection, while death occurred in one chicken at 6 days post-infection. Clinical signs observed in the infected group are swollen eyelids, lethargic, and decreased appetite. After 6 days post-infection, some chickens slowly recovered, until it exhibited no clinical symptoms.

**Figure 4:**
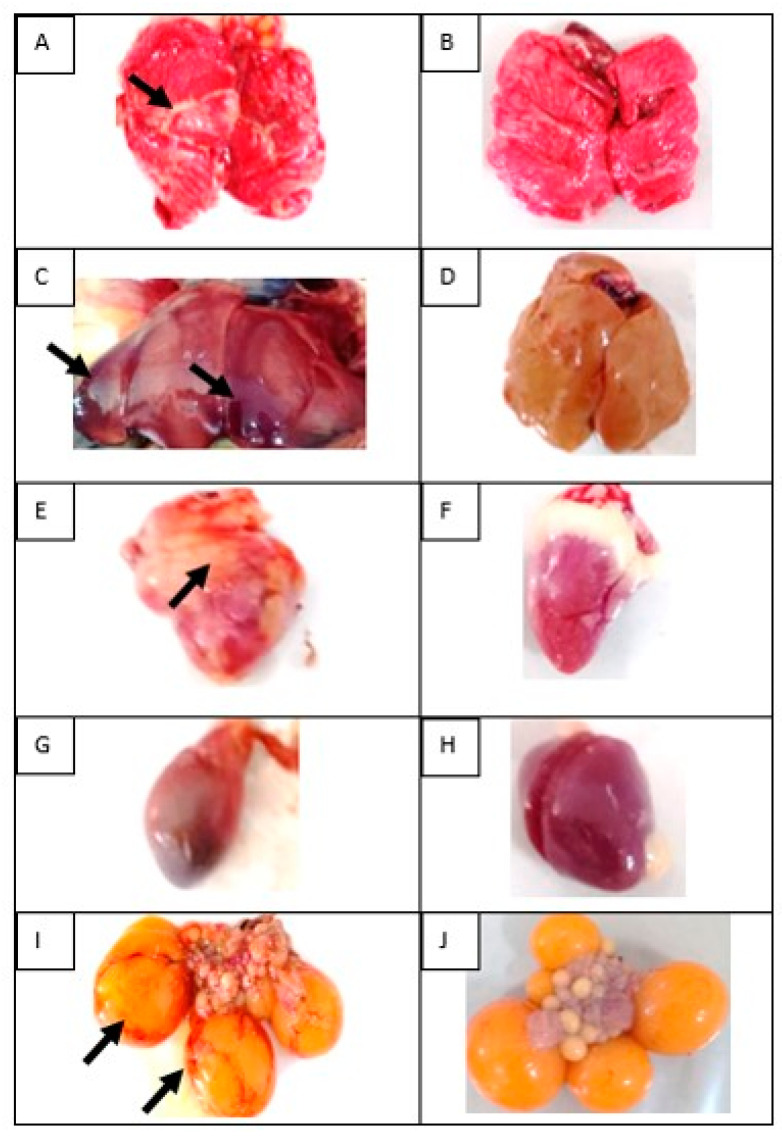
Gross lesions in multiple organs of infected chicken. (**A**) Lung: congested, white to yellow exudate covered the surface of lungs (black arrow); (**C**) Liver: enlarge, congested in some areas (black arrow), blunted, and brittle; (**E**) Heart: enlarged, petechiae to purpura in the surface of organs (black arrow); (**G**) Spleen: atrophy; (**I**) Ovarian follicles: congested (black arrow); (**B**,**D**,**F**,**H**,**J**) Normal organs of lung, liver, heart, spleen, and ovarian follicles, respectively.

The pathology anatomy changes in dead SPF chickens are shown in Figure 4. The lung was congested, while the liver appeared enlarged, hemorrhagic, and brittle. Ovarian follicles showed an enlargement of blood vessels, the spleen organs experienced atrophy, and the heart was enlarged.

**Figure 5:**
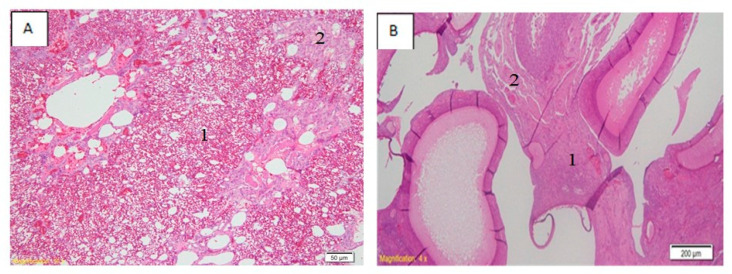
Histopathological lesions in lungs (**A**) and ovaries (**B**). Accumulation of fluid and hemorrhagic in lungs (**A.1**), as well as an infiltration of mononuclear cells (**A.2**). Mononuclear cell infiltration (**B.1**) and hemorrhagic (**B.2**) also occurred in ovaries.

A description of the histopathological lesions in the lungs and ovaries of chickens infected with BLi25Ut/18 virus is presented in Figure 5. The lungs undergo edema, hemorrhagic (Figure 5(A.1)), and mononuclear cell infiltration (Figure 5(A.2)). The ovaries showed mononuclear cell infiltration (Figure 5(B.1)) and hemorrhagic (Figure 5(B.2)).

**Figure 6:**
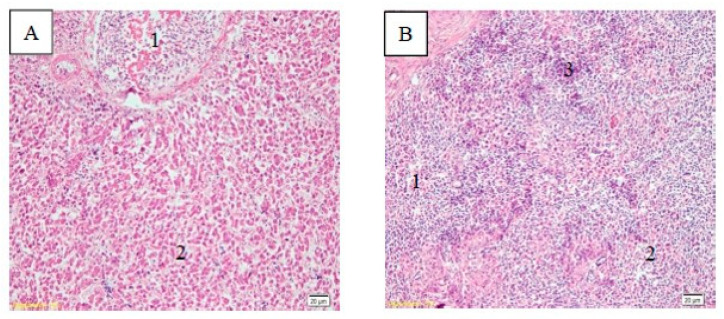
Histopathological changes in the liver (**A**) and spleen (**B**). The liver underwent hepatocyte necrosis and the infiltration of mononuclear cells (**A.2**); (**A.1**) is the portal tract; in the spleen, the white pulp area (**B.1**) shows necrosis and vacuolization (**B.2**), as well as lymphocyte cells accumulation (**B.3**).

The histopathological changes in the liver and spleen of infected chicken are presented in Figure 6. The liver was necrotic with mononuclear cell infiltration, whereas the spleen experienced necrosis and lymphocyte cells accumulation.

**Figure 7:**
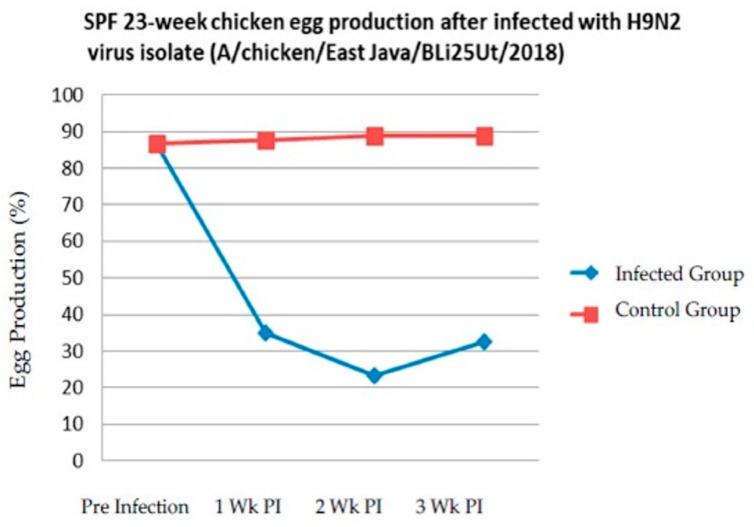
Graphic of decreased egg production in SPF chickens, PI: Post-Infection; Wk: Week(s).

SPF chicken’s egg production is shown in Figure 7. A very significant decreased in egg production occurred at 14 days-post infection.

Initially before being infected with BLi25Ut/18 virus, egg production reached 86.33%; then, it decreased sharply to 35% at one week post-infection. At two weeks post-infection, egg production continued to decrease to 23.21%. At three weeks post-infection, egg production was slightly increased up to 32.63%. The total decrease of egg production for two weeks post-infection was 63.12%. At the end of study, the mortality reached 10% in chickens infected with BLi25Ut/18 virus.

### 3.3. Vaccine Efficacy against BLi25Ut/18 Virus

**Figure 8:**
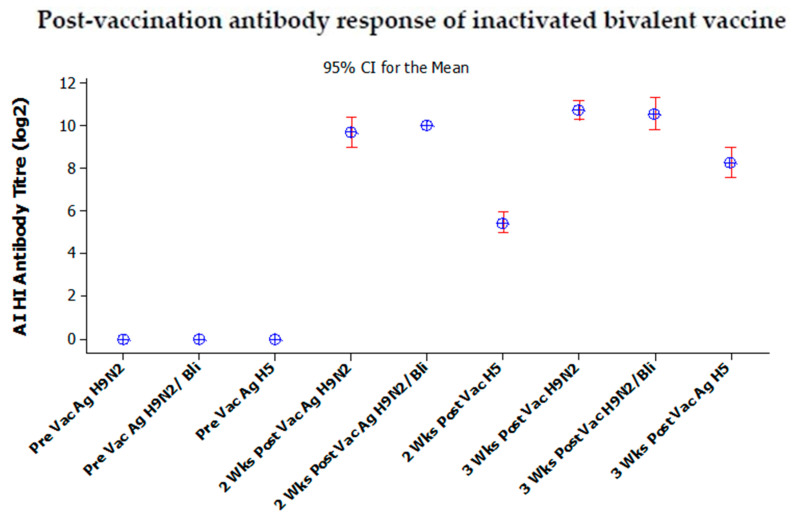
The average antibody titer in post-vaccination SPF chickens with inactivated bivalent vaccine. Vac: Vaccination; Wks: Weeks; Ag: Antigen; H9N2: H9N2/2017; H9N2/Bli: BLi25Ut/18; H5: H5N1/2013.

**Figure 9:**
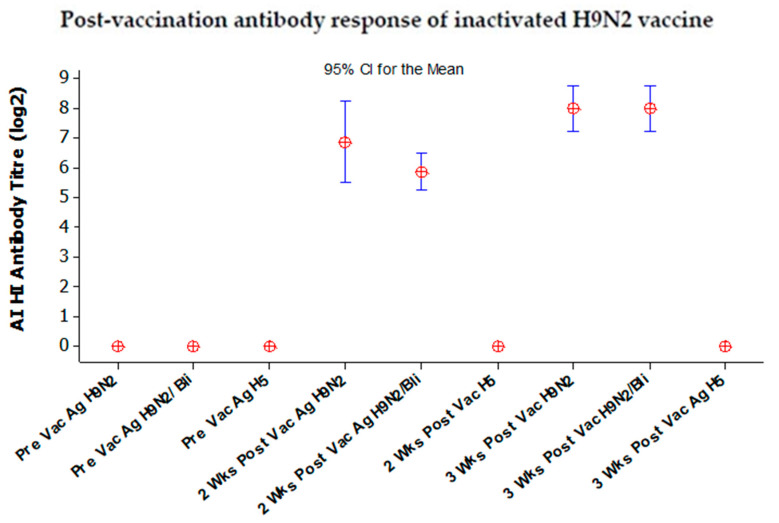
The average antibody titer in post-vaccination SPF chickens with inactivated monovalent H9N2 vaccine. Vac: Vaccination; Wks: Weeks; Ag: Antigen; H9N2: H9N2/2017; H9N2/Bli: BLi25Ut/18; H5: H5N1/2013.

The antibody responses of chickens after vaccination using inactivated bivalent and monovalent vaccines are shown in Figure 8 and Figure 9, respectively.

The antibody post-vaccination response in Group 1, using bivalent vaccine, is presented in Figure 8. The results showed that the average antibody titer against AI H9N2/2017, BLi25Ut/18 and H5N1/2013 antigens are 9.71 log2 (CI 9.01–10.41), 10 log2, and 5.42 log2 (CI 4.93–5.92), respectively after two weeks post-vaccination. Furthermore, in the three weeks after vaccination, the antibody titer increased sequentially, namely 10.71 log2 (CI 10.26–11.16), 10.5 log2 (CI 9.84–11.29), and 8.28 log2 (CI 7.58–8.98) against H9N2/2017, BLi25Ut/18, and H5N1/2013 antigens, respectively.

The antibody post-vaccination response of Group 2, chickens vaccinated with the inactivated monovalent H9N2 vaccine, is given in Figure 9. The results of the average antibody titer against the AI H9N2/2017, homolog BLi25Ut/18 and H5N1/2013 antigens are 6.85 log2 (CI 5.5–8.21), 5.85 log2 (CI 5.21–6.49), and 0, respectively, after two weeks post-vaccination. After three weeks post-vaccination, the antibody titer increases sequentially, namely 8 log2 (CI 7.24–8.75), 8.3 log2 (7.24–8.75), and 0 against the H9N2/2017, BLi25Ut/18, and H5N1/2013 antigens, respectively.

**Figure 10:**
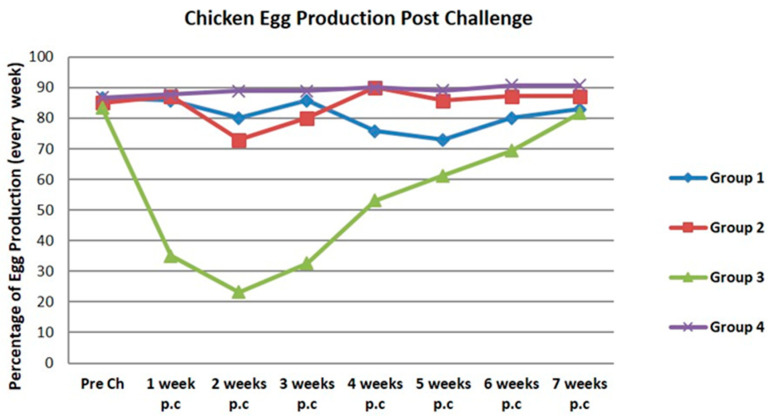
Egg production graphic of vaccinated and control group challenged with BLi25Ut/18 virus, observations were carried out for seven weeks after the challenge. Ch: challenge; p.c: post-challenge.

The group of vaccinated chickens using the bivalent vaccine (Group 1) showed a 6.67% reduction of egg production in two weeks post-challenge. Before being challenge with the BLi25Ut/18 virus, the egg production was 86.67%. Then, the egg production decreased to 80% after two weeks post-challenge. The eggs production started to increase to more than 80% after 6–7 weeks post-challenge (Figure 10). The results in the vaccinated chickens in Group 2, which received the monovalent H9N2 vaccine, showed 12.14% reduction of egg production after two weeks post-challenge. Initially egg production was 85% and decrease down to 72.86% in two weeks post-infection. The egg production started to increase in 4 weeks post-challenge. Group 3, without vaccination, showed 60.12% reduction of egg production (i.e., from 83.33% to 23.21%) in two weeks post-challenge. Group 4 as a control that was not challenge had stable egg production ranging from 86.67% to 90.71% during 7 weeks of observation. Statistical analysis showed that the decrease of egg production at 2 weeks post-challenge was significantly different between groups (*p* Value < 0.05). The results of the challenge test from the parameters of egg production showed that the vaccinated chicken groups gradually increased its egg production by ≥80% ranging from 4 to 7 weeks after challenge.

Virus shedding in Groups 1–3 that were challenged with BLi25Ut/18 virus is presented in Table 3. Virus shedding was found until 8 days post-infection and was not found at 14 days post-infection in Groups 1 and 2 (Table 3). In Group 3, virus shedding of BLi25Ut/18 virus was found until 14 days post-infection.

The vaccine efficacy result showed a decrease in egg production in the two weeks post-treatment of only 6.67% in Group 1 and 12.14% in Group 2. The vaccinated chickens showed an increased in egg production to more than 80% ranging from 4 to 7 weeks post-challenge and prevented death due to BLi25Ut/18 virus challenge, as well as reduced virus shedding.

## 4. Discussion

The LPAI H9N2 virus has unique characteristics, among other LPAI viruses. H9N2 viruses are capable of infecting a variety of host species, including humans, and they can become genetic material donors for other influenza viruses. Co-circulation between H9N2 viruses and circulating viruses in the field, such as HPAI H5N1 virus, can cause reassortment resulting in novel viruses with higher pathogenicity [18,19].

Incidents of reassortant viruses between H5N1 and H9N2 viruses have been reported in several publications. In 2013, it was reported that the H5N1 virus acquired the PB1 gene from the H9N2 virus in Bangladesh. Based on the intravenous pathogenicity index in SPF chickens, this reassortant virus belongs to the HPAI virus [24]. Then, in 2014, two H5N1 viruses isolated from the live poultry market underwent a reassortant with H9N2 virus. Two AI viruses H5N1 clade 2.3.2.1 carried the M gene from the H9N2 virus, which was isolated in 2013 [25]. In 2014, the H5N2 virus isolated from chickens on the live poultry market in Vietnam had HA, NP, PA, and M genes, which were phylogenetically close to the H5N1 virus clade 2.3.2.1, but the NA, PB1, and NS genes had genetic relationship with the H9N2 virus. The reassortant H5N2 virus also belongs to the HPAI virus because it has several of the basic amino acids in HA1 and is capable of causing death in experimental animals [26].

In 2015, three reassortant H5N2 viruses were isolated from chicken farms in China. The H5N2 viruses obtained HA and M genes from the H5N1 virus, while the other genes were obtained from the H9N2-like virus [27]. A reassortment incident between the H5N1 and H9N2 viruses from AI outbreaks in wild birds was reported in the same year. Genetic analysis showed that the reassortant virus was an HPAI H5N1 clade 2.3.2.1c that obtained the PB2 gene from the LPAI H9N2 virus originating from Asia [28].

A case of infection by the novel H5N1 clade 2.3.2.1c virus in ducks and turkeys in India was reported in 2014. Genetic analysis of the H5N1 clade 2.3.2.1 virus showed the presence of the PB2 gene from the H9N2 virus [29]. Reassortant between the H5N1 clade 2.3.2.1c and H9N2 viruses have also previously been reported in China, Nigeria, and Dubai [28,30,31]. In 2018, a reassortant virus was reported in the live poultry market in Cambodia; the H5N1 clade 2.3.2.1c virus obtained the PB1 gene from the H9N2 virus [32].

Reassortant events between H5N1 and H9N2 viruses also occurred in Indonesia, as well as in this study. Phylogenetic analysis showed that the two H9N2 isolates namely A/chicken/East Java/BLi25Ut/2018 and A/chicken/Central Java/SLO.105/2018 were reassortant H9N2 viruses, with the HA, NA, M, NS, PA, NP, and PB1 genes derived from H9N2 virus, while the PB2 gene was derived from the H5N1 virus. The mechanism of genetic material exchange in reassortment between influenza virus strains is not yet fully known, and it is still unclear where it occurs. This may occur in the nucleus during replication or when RNA secretes from the nucleus to the cell membrane, in the cytoplasm of the cell after the release of the RNA progeny segment, or during virus assembly [33]. The reassortment process may be controlled by certain signal differences in initiating RNA segment packaging. The optimal conditions for reassortment and segment changes can be influenced by virus strains, but it is not yet certain what kind of factors control the reassortment. Reassortment can occur in the process of assembling a virus in which errors in segment combinations occur because the system cannot distinguish the origin of the RNA gene segment from the two subtypes of the virus [34,35]. The reassortment process can change the biological character of influenza viruses significantly, as in the case of LPAI H7N9 subtype infection, which can cause human infection in China in 2013 [36]. Meanwhile, reassortment between H9N2 and H1N1pdm2009 can cause increased virulence in mice, increased transmissibility in ferrets and pigs, and increased replication in pigs. Reassortant between H9N2 and H1N1pdm2009 is considered to have the risk of infecting humans if reassortant events in pigs continue to occur [37].

In this study, it was reported that chicken infected with the reassortant H9N2 virus experienced various clinical signs such as decreased appetite, lethargy, decrease of egg production, and moderate to severe lesion in multiple organs. Outbreak events in the field and our recent data showed that the mortality rate caused by the reassortant H9N2 virus was higher than that of wt-H9N2 virus infection. Our previous data reported that no mortality was observed from wt-H9N2 infection [22]. In another study, it was shown that the mortality rate caused by wt-H9N2 virus infection was varied from >1–2% to 5% [13]. Thus, we considered that the 10% (1 out of 10) mortality rate resulted by reassortant H9N2 virus infection in this study might be caused by the PB2 gene obtained from the H5N1 virus. Several amino acid residues from the PB2 gene are known to affect the pathogenicity and virulence by increasing the polymerase activity. This showed that the presence of internal reassortant genes originating from the H5N1 virus in the H9N2 virus might cause the LPAI virus to become more pathogenic [37].

Aside from causing an increase in virulence and efficiency in pathogenicity and transmission, several reassortment events were found to cause attenuation in the reassortant virus. This event was reported in Indonesia in 2018. Reassortment between the HPAI H5N1 and the LPAI virus created a new virus with a different character, which was only able to cause viral infections in the nose and lung tissue; this indicated that the viruses were able to infect mice without further adaptation but were unable to cause systemic disease. The two viruses showed attenuation to replicate in poultry compared to non-reassortant viruses. The attenuation to infect both the avian and mammalian systems shows that the acquisition of internal genes from the LPAI virus is sufficient to reduce the replication ability despite the multibasic cleavage of HA protein being present [7].

In connection with the reassortment incident, several commercial poultry vaccines that feature a combination of LPAI and HPAI are used in several countries to protect the poultry industry and reduce the risk of human infection through poultry. However, they do not have good effectiveness due to the rapid mutations of the AI virus. Circulation of the reassortant H9N2 virus in the field certainly requires an effective vaccine in controlling the disease. Vaccination is an alternative control program that is quite effective for AI disease [38,39]. Vaccination can be a good approach to support the eradication program if combined with other appropriate control methods [40]. Vaccination can play an important role in controlling, preventing, and eliminating AI viruses. In areas that are endemic to AI, vaccination can reduce the risk of more severe disease in poultry and reduce the amount of virus released into the environment to reduce exposure to humans and the possibility of zoonotic influenza [41].

The results indicated that the monovalent and bivalent vaccine could induce an antibody response in vaccinated chickens against the reassortant H9N2 virus. This study showed a minimum decrease in egg production of only 6.67% at two weeks post-challenge in the vaccinated group using the bivalent vaccine and 12.14% in the vaccinated group using the monovalent H9N2 vaccine as well as reduced virus shedding compared to the unvaccinated group. The vaccinated group’s egg production started to increase by more than 80% ranging from 4 to 7 weeks post-challenge. These results are in line with the development of vaccines that use a combination between LPAI (H9N2) and HPAI (H5N1 and H5N8) in Egypt that can improve the humoral immune response and provide full protection against the H9N2, H5N1, and H5N8 viruses. The efficacy of this vaccine is considered able to reduce the cost of vaccination and is effective to use in the field [42]. In addition to vaccination, prevention strategies can be carried out by conducting routine surveillance to monitoring the evolution and presence of AI viruses in poultry as well as to anticipate the emergence of AI viruses that are more adaptable to humans and development of future vaccine.

## 5. Conclusions

Reassortant H9N2 virus infection cause clinical signs such as decrease appetite, lethargy, moderate to severe lesion in multiple organs, decrease of egg production, and mortality rate of up to 10% (1 out of 10). The bivalent and monovalent vaccines that were used in this study can induce immune response in vaccinated chickens and good protection efficacy against reassortant virus by reducing virus shedding, mortality, clinical signs, and decreased egg production.

## Figures and Tables

**Table 1:** Results of Hemagglutination Inhibition (HI) assay using specific sera to confirm that BLi25Ut/18 isolate has no contamination with other viruses.

Antisera	BLi25Ut/18
H9N2/2017	10 log2
H5N1 Clade 2.1.3	Negative
H5N1 Clade 2.3.2	Negative
ND genotype VII	Negative
ND genotype VI	Negative
ND RIVs genotype 2	Negative

**Table 2:** The pathogenicity test results of BLi25Ut/18 virus on specific pathogen-free (SPF) chickens aged 23 weeks.

Post-Infection (Days)														
Group	1	2	3	4	5	6	7	8	9	10	11	12	13	14
Infected	h:10	h:10	h:10	s:10d	s:10a,d	s: 9d dt:1	s:8d h:1	s:6d h:3	s:6d h:3	s:6d h:3	s:6d h:3	s:6d h:3	s:6d h:3	s:6d h:3
Control	h:10	h:10	h:10	h:10	h:10	h:10	h:10	h:10	h:10	h:10	h:10	h:10	h:10	h:10

Note: s = sick (lethargic, decreased appetite); a = one of the chicken’s eggshell was soft; d = decrease of egg production; h = healthy; dt = death.

**Table 3:** Re-isolation of BLi25Ut/18 virus challenge from allantoic fluid of 10-day-old embryonated chicken eggs to detect virus shedding in SPF chickens.

Challenge Group	Virus Shedding in SPF Chickens (Day Post-Challenge)		
	Days 4	Days 8	Days 14
Group 1. Inactivated bivalent vaccine	3/10	2/10	0/10
Group 2. Inactivated monovalent H9N2 reassortant vaccine	2/10	1/10	0/10
Group 3. Unvaccinated SPF chickens	9/10	4/9 *	2/9 *

Notes: * reduction number due to mortality.

## Supplementary Materials

The following are available online at https://www.mdpi.com/2076-393X/8/3/449/s1. DNA sequence Primers.
