# Supplementary material for: Vaccine Efficacy on the Novel Reassortant H9N2 Virus in Indonesia
Source: Vaccines (Basel). 2020 Aug 10;8(3):449. doi: 10.3390/vaccines8030449 (PMC7565121; doi:10.3390/vaccines8030449)
Supplement: Supplementary file 1 [file vaccines-08-00449-s001.pdf]

DNA sequence Primers for H9N2 subtype

H9HA69F1	5'-ATCTGCATCGGCTACCAATC-3'
H9HA740F2	5'-CAGGTCAGACATTGCGAGTA-3'
H9HA836R1	5'-GTCTTCAGGATTCTTCCGTG-3'
H9HA1472R2	5'-CGAATTGTCTCCATGCACTG-3'
H9Matr_7F	5'-CGCAGAGGCTTGAAGATGTC-3'
H9Matr_955R	5'-ACCATCGTCAACATCCGCAG-3'
H9NA-352F	5'-TATGTATCGTGCGGTCTTGG-3'
H9NA-1288R	5'-CCTGTGGTCTTCCTCTTATC-3'
H9NP_21F	5'-CTGAGTGACATTCACGCCAT-3'
H9NP_406R	5'-CGAACAATGGAGAAGACGCA-3'
H9NP_1352R	5'-GGATGTTCTGCCTTCAGTGT-3'
H9NS-6F	5'-CCAACACTGTGTCAAGCTTC-3'
H9NS-683R	5'-CTGGCTCAATTGTTCTCTCC-3'
H9PA-254F	5'-CAATGGCATGGACAGTAGTG-3'
H9PA_809R	5'-TTCAGAGGACGTGGTGTTGT-3'
H9PB1_93F	5'-CCATGGAACAGGAACAGGAT-3'
H9PB1_678R	5'-TGTGTTCAGTGTCAGTGCTC-3'
H9PB2_1090F	5'-GCAACAGCTATCCTGAGGAA-3'
H9PB2_2057R	5'-CTTAGTACCGCAGACTCCAC-3'

H9HA69F1	5'-ATCTGCATCGGCTACCAATC-3'
H9HA740F2	5'-CAGGTCAGACATTGCGAGTA-3'
H9HA836R1	5'-GTCTTCAGGATTCTTCCGTG-3'
H9HA1472R2	5'-CGAATTGTCTCCATGCACTG-3'
H9Matr_7F	5'-CGCAGAGGCTTGAAGATGTC-3'
H9Matr_955R	5'-ACCATCGTCAACATCCGCAG-3'
H9NA-352F	5'-TATGTATCGTGCGGTCTTGG-3'
H9NA-1288R	5'-CCTGTGGTCTTCCTCTTATC-3'
H9NP_21F	5'-CTGAGTGACATTCACGCCAT-3'
H9NP_406R	5'-CGAACAATGGAGAAGACGCA-3'
H9NP_1352R	5'-GGATGTTCTGCCTTCAGTGT-3'
H9NS-6F	5'-CCAACACTGTGTCAAGCTTC-3'
H9NS-683R	5'-CTGGCTCAATTGTTCTCTCC-3'
H9PA-254F	5'-CAATGGCATGGACAGTAGTG-3'
H9PA_809R	5'-TTCAGAGGACGTGGTGTTGT-3'
H9PB1_93F	5'-CCATGGAACAGGAACAGGAT-3'
H9PB1_678R	5'-TGTGTTCAGTGTCAGTGCTC-3'
H9PB2_1090F	5'-GCAACAGCTATCCTGAGGAA-3'
H9PB2_2057R	5'-CTTAGTACCGCAGACTCCAC-3'
